# Carotid Artery Reconstruction with an Autologous Bifurcated Saphenous Vein Graft

**DOI:** 10.3400/avd.nmt.23-00118

**Published:** 2024-03-15

**Authors:** Tomohiro Tsunekawa, Ryo Utakata, Yukiomi Fukumoto, Tomitaka Kubo, Fumiya Kuze

**Affiliations:** 1 Department of Cardiovascular Surgery, Central Japan International Medical Center, Minokamo, Gifu, Japan; 2 Department of Otorhinolaryngology, Central Japan International Medical Center, Minokamo, Gifu, Japan

**Keywords:** carotid artery, vascular surgery, autologous vascular graft

## Abstract

We present a new technique for carotid artery reconstruction using a modified bifurcated saphenous vein graft in a patient with a malignant neck tumor. This technique can optimize the size match between the SVG and common carotid artery, as well as the internal and external carotid arteries. Post operative computed tomography performed a year after the operation demonstrated excellent graft alignment and patent carotid arteries.

## Introduction

We herein report our technique for internal and external carotid artery reconstruction using a modified bifurcated saphenous vein graft (SVG) in a patient with a malignant neck tumor. Informed consent for patient information and images to be published was obtained from the patient.

## New Methods

**Figure 1:**
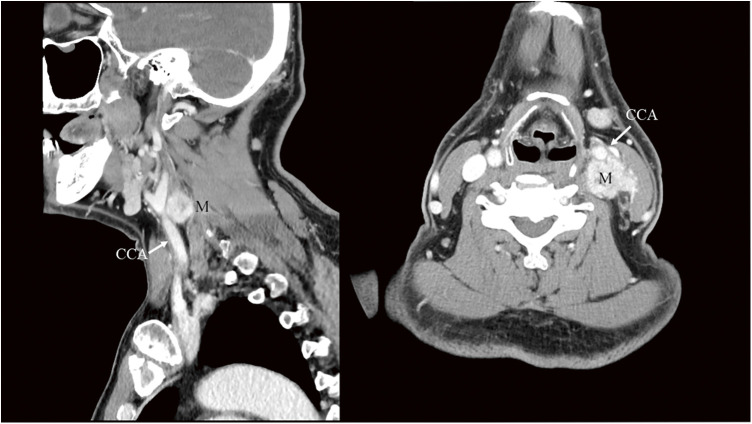
Fig. 1 Sagittal and axial planes of preoperative enhanced computed tomography. M: left cervical mass; CCA: left common carotid artery

A 58-year-old man presented to the outpatient department of otorhinolaryngology with a left-sided cervical mass increased in size. His medical history was unremarkable, except for hypertension. A needle biopsy of the mass revealed that it was a metastatic tumor of the cervical lymph node originating from a thyroidal papillary adenocarcinoma. Enhanced computed tomography (ECT) suggested that the mass had invaded the left carotid arteries (**Fig. 1**). The patient was referred to our department, and concomitant resection and reconstruction of the left carotid arteries during the removal of the tumor was considered appropriate. A preoperative carotid clamp test was performed to evaluate the tolerance of intraoperative clamping of the left common carotid artery (CCA). Briefly, the left CCA was occluded by a balloon catheter for 5 minutes. During clamping, the mean stump pressure of the left CCA decreased to 70% of the pre-clamp blood pressure. Angiographically, the left internal carotid artery (ICA) was well perfused by the right ICA through the anterior communicating artery. Blood supply from the vertebrobasilar circulation through the posterior communicating artery was poor. Brain perfusion scintigraphy using hexamethylpropylene amine oxime, which was injected during the clamp test, showed a 5% decline in the accumulation on the left side of the brain compared with the right side. During the clamp test, the patient did not present any neurological symptoms. Based on these results, the patient was considered to be able to tolerate simple clamping of the left CCA during the procedure.

ECT showed the diameters of the common, internal, and external carotid arteries (CCA, ICA, and ECA, respectively) to be 8.9 mm, 5.6 mm, and 5.1 mm, respectively. After resection of the left lobe of the thyroid gland, the cervical mass was carefully dissected from surrounding tissues. The mass tightly adhered to the bifurcation of the carotid arteries. The left carotid artery was then removed and reconstructed.

**Figure 2:**
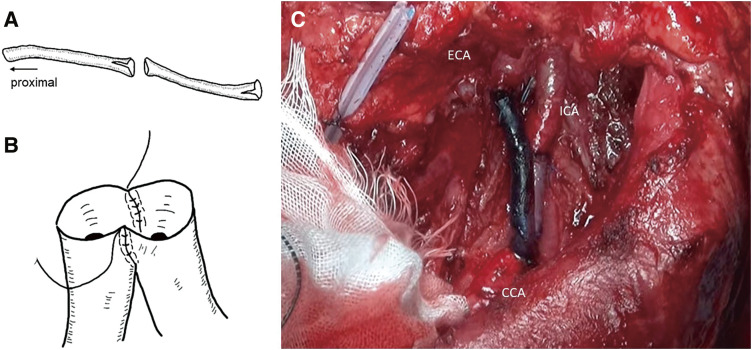
Fig. 2 (**A** and **B**) Schematic illustration of preparing the saphenous vein graft. (**C**) Operative view of carotid artery reconstruction with self-manufactured bifurcated saphenous vein graft. CCA: common carotid artery; ICA: internal carotid artery; ECA: external carotid artery

The SVG was harvested 12 cm from the right thigh. The mean SVG diameter was 4 mm. The SVG is divided into two segments at the center of its length. A 1-cm-long slit was made at the distal end of both segments (**Fig. 2A**). The trunk of the bifurcated SVG was created by sewing the slits together. Each edge of the slit was anchored to the facing edge of the other slit using a single stitch of 6-0 polypropylene suture. The slits were then sewn together with a continuous over-and-over suture. Attention must be paid to avoid placing the suture knots inside the vascular lumen to prevent thrombus formation at the suture line. The created trunk of the bifurcated SVG was 5 mm in length (**Fig. 2B**). CCA, ICA, and ECA were cross-clamped and resected with sufficient margins from the edge of the tumor. The tissue oxygenation index (TOI) of both sides of the brain was continuously monitored during the procedure using NIRO 200NX (Hamamatsu Photonics, Hamamatsu, Japan). Generally, if TOI shows a >20% decline from the baseline value, we routinely use distal perfusion devices such as shunt tubes.

Vein grafting was performed consistently in a reverse fashion. The reversed SVG did not undergo a valvulotomy. The trunk of the bifurcated graft, measuring 8 mm in diameter, was anastomosed to the common carotid artery using continuous 5-0 polypropylene sutures in an end-to-end fashion. One of the distal ends of the bifurcated graft was anastomosed to the ICA by using continuous 6-0 polypropylene sutures in an end-to-end fashion. The internal carotid artery (ICA) cross-clamp time was 20 min. ECA reconstruction was performed in the same manner as the ICA reconstruction (**Fig. 2C**). TOI showed no significant decline during the cross-clamp, and no distal perfusion devices were required.

**Figure 3:**
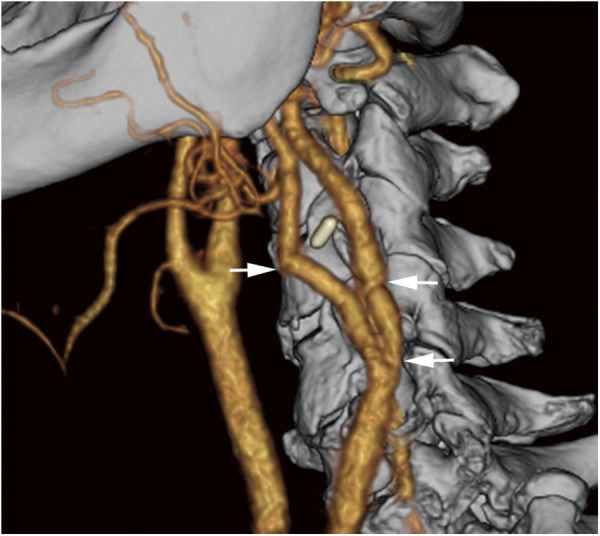
Fig. 3 A three-dimensional view of postoperative enhanced computed tomography findings obtained one year after the operation. The white arrows indicate the anastomosis site.

Mechanical ventilation was discontinued on postoperative day (POD) 1 without any neurological complications. Low-dose aspirin was administered on POD 1, which was continued for 6 months. The patient was discharged on POD 10. Post operative ECT performed a year after the operation demonstrated excellent graft alignment and patent carotid arteries (**Fig. 3**). No size mismatch was observed at the anastomosis site between the graft and carotid arteries.

## Discussion

One major issue related to carotid artery reconstruction is the graft material to be used. Our preference for autologous SVG is based on its superior resistance to infection.^1^^–^^3)^ We originally utilized the bifurcated SVG as a vascular graft for reconstruction of the femoral arterial bifurcation, especially in patients with either a synthetic graft infection or an infective pseudoaneurysm of the femoral arteries. This is the first case in which the technique was applied to carotid artery reconstruction. Illuminati et al. reported the excellent long-term patency of ePTFE.^4^^,^^5)^ However, while ePTFE grafts offer better resistance to tissue scarring and radiation therapy than autologous tissue grafts, they have the inherent disadvantage of vulnerability to infection.

One of the major advantages of our new method is that we can double the diameter of the proximal end of the SVG and adjust the mismatch between the diameters of the SVG and CCA. At the same time, the distal ends of the bifurcated SVG remained at the normal diameter, which matched the diameter of the ICA and ECA. Size mismatch between the SVG and the native carotid arteries can act as a major factor influencing SVG graft stenosis or occlusion. Flow turbulence causes shear stress at the size-mismatched anastomosis site, which can produce intimal abrasion and lead to intimal inflammatory changes. Some authors have reported that the use of large-caliber autologous grafts, such as deep or superficial femoral veins, for carotid reconstruction can prevent graft thrombosis or occlusion.^2^^,^^6)^ Our bifurcated SVG can provide twice the diameter at the proximal end of the SVG without any complicated techniques and allows for better size matching with the CCA in this case. However, in our method, the suture line used to create the bifurcation crossed the proximal anastomosis line. This may increase the risk of thrombus formation at the proximal anastomosis site. Low-dose aspirin was administered for 6 months postoperatively to avoid thrombus adhesion at the suture line. Special attention should be paid to prevent placing suture knots inside the vascular lumen.

The benefits of ECA preservation remain unclear.^1^^,^^7)^ Although most surgeons agree to sacrifice the ECA, we recommend reconstructing the ECA whenever possible. The ECA can provide important collateral flow to both the brain and eye in the face of ipsilateral ICA occlusion. In addition, if the ECA is sacrificed, emboli may originate from the external carotid stump, which can cause ophthalmologic or neurological symptoms.^8)^ An additional advantage of ECA reconstruction is the possibility of using this vessel as the arterial supply for free tissue transfer, especially in patients with malignant tumors who will experience extensive tissue loss if they develop recurrent disease.^1)^

Our bifurcated SVG technique is a simple but effective option for ICA and ECA reconstruction in patients with advanced neck cancer. This technique can optimize the size match between the SVG and CCA, as well as between the SVG and ICA or ECA.

## Funding

This research received no specific grants from any funding agency in the public, commercial, or not-for-profit sectors.

## Research Ethics

The institutional review board of Central Japan International Medical Center waived approval.

## Informed Consent

Written informed consent for the publication of patient information and images was provided by the patient.

## Disclosure Statement

All authors have declared no conflict of interest.

## Author Contributions

Study conception: TT

Data collection: TT and RU

Manuscript preparation: TT

Clinical review and revision: all authors

Final approval of the article: all authors

Accountability for all aspects of the work: all authors.
